# Global Dynamics of an Avian Influenza A(H7N9) Epidemic Model with Latent Period and Nonlinear Recovery Rate

**DOI:** 10.1155/2018/7321694

**Published:** 2018-02-22

**Authors:** Rui Mu, Youping Yang

**Affiliations:** School of Mathematics and Statistics, Shandong Normal University, Jinan 250014, China

## Abstract

An SEIR type of compartmental model with nonlinear incidence and recovery rates was formulated to study the combined impacts of psychological effect and available resources of public health system especially the number of hospital beds on the transmission and control of A(H7N9) virus. Global stability of the disease-free and endemic equilibria is determined by the basic reproduction number as a threshold parameter and is obtained by constructing Lyapunov function and second additive compound matrix. The results obtained reveal that psychological effect and available resources do not change the stability of the steady states but can indeed diminish the peak and the final sizes of the infected. Our studies have practical implications for the transmission and control of A(H7N9) virus.

## 1. Introduction

Avian influenza A(H7N9) is a subtype of influenza viruses that have been detected in birds and confirmed to be low pathogenic among poultry in the past [1]. Human infections by this particular A(H7N9) virus had not previously been reported until it was found in March, 2013 in China (WHO). It appears that A(H7N9) virus has become a highly pathogenic virus for human species who directly or indirectly contacts poultry carrying virus [2, 3]. From September 1, 2016, to April 31, 2017, 643 cases of avian influenza A(H7N9) laboratory-confirmed cases have been reported in Mainland China, including 233 cases that have died (China CDC), which imposes a serious threat to public health.

There are different types of models to analyze the dynamical behavior of avian influenza virus and assess useful control measures. Iwami et al. [4] showed that when mutant avian influenza had already occurred, reducing the contact rate of susceptible with infectious humans may have a positive effect on preventing the second outbreak. Liu and Fang [5] formulated a two-host model to investigate the impact of screening and culling of infected poultry. Liu et al. [6] considered different growth laws of the avian population, to present that the necessary and sufficient condition for periodic solution existing is the Allee effect in avian population. However, most of these models ignore the latent period between infection and symptom onset in human populations, which does exist on the basis of the reported infection cases. Hence, we introduce the incubation period into our model to further study the internal transmission mechanism of A(H7N9) virus.

When a disease breaks out, people's awareness of its severity can generate a profound psychological impact on the individuals' behaviors to reduce unnecessary contact with infections [7]. Wang et al. [8] found that 77% of urban respondents in their investigation reported that they visited live markets less often after influenza A(H7N9) cases were first identified in China in March 2013. Wu et al. [9] showed that, in the second wave of avian influenza A(H7N9), greater worry among respondents led to changes in protective behaviors such as less visit to live poultry markets and less purchase of live poultry. To model the reduction in contacts due to the psychological effect, various incidence rates were formulated by researchers [10–13]. In this paper, we will modify these functions to investigate the psychological effect on the transmission of A(H7N9) virus.

In previous dynamic models of avian influenza A(H7N9), one usually assumed the recovery rate as a constant, which means that the treatments were always sufficient. But in fact, hospital resources (such as doctors, drugs, hospital bed, and isolation places) are limited to public, especially when a disease breaks out [14]. According to reported cases by CDC, human infections with A(H7N9) virus and common flu virus have similarities in infected time and the early clinical manifestations; therefore, some available hospital resources have already been occupied. Hospital bed-population ratio, the number of available hospital beds per 10,000 population, is widely used by health planners as a method of estimating resource availability to the public [15]. Abdelrazec et al. [16] established a model for the transmission dynamics of dengue fever, with the recovery rate function reflected by the hospital bed-population ratio and the number of infections. They found that both the oscillations and backward bifurcation occur attributed to limited hospital resource capacity. And that the basic reproduction ratio *R*_0_ is not enough to determine whether the disease eliminates or not. In this paper we will introduce the recovery rate function to explore the impact of available hospital resources in spreading A(H7N9) virus.

This paper is organized as follows. In Section 2, we formulate the A(H7N9) transmission model incorporating the combined impact of psychological effect and available hospital resources. The dynamical behavior of the model is investigated in Section 3, including the existence and global stability of equilibria. In Section 4, we carry out numerical simulations to verify the theoretical results, and the conclusions and discussions for further work are presented in Section 5.

## 2. Model Formulation

Based on information reported, there is no evidence of sustained human-to-human transmission, although there have been two family clusters reported. Thus, we always assume the transmission of A(H7N9) virus is not from person to person. In our model, we divide the poultry into two subclasses: susceptible (*S*_*p*_) and infectious (*I*_*p*_), respectively, and the human population is divided into four subclasses: susceptible (*S*_*h*_), latent (*E*_*h*_), infectious (*I*_*h*_), and recovered (*R*_*h*_). Before constructing the model, we make the following assumptions.

(i) Taking into account the factors such as poultry market mobility, environment capacity, and the existing populations, the susceptible poultry is subject to the logistic growth [6]

(1) $$\begin{array}{c} r_{p}S_{p}\left(\;1-\frac{S_{p}}{K_{p}}\;\right), \end{array}$$

where *r*_*p*_ and *K*_*p*_ are the intrinsic growth rate and maximal carrying capacity of the poultry.

(ii) Due to psychological effect, the infection force may decrease when the number of infectious individuals increases. Hence, we modify a nonlinear incidence rate proposed by Liu et al. [17] to describe the transmission of the virus from infected poultry to susceptible individuals, with the following form:

(2) $$\begin{array}{c} \beta_{h}S_{h}\frac{I_{p}}{1+aI_{h}}, \end{array}$$

where *β*_*h*_ is the transmission coefficient, *β*_*h*_*I*_*p*_ measures the infection force of the disease, *a* is a nonnegative constant, and 1/(1 + *aI*_*h*_) measures the inhibition due to the psychological effect.

(iii) We assume that latent humans (*E*_*h*_) do not take up the hospital bed resources during the latent period and, meanwhile, consider the impact of hospital resources on the recovery rate, first proposed by Shan and Zhu [18], which can be expressed in the following formula:

(3) $$\begin{array}{c} \mu \left(\;b,I_{h}\;\right)=\mu_{0}+b\frac{\left(\mu_{1}-\mu_{0}\right)}{b+I_{h}}, \end{array}$$

with

(4) limb→+∞⁡μb,Ih=μ1,limIh→0+⁡μb,Ih=μ1,limIh→+∞⁡μb,Ih=μ0,limb→0+⁡μb,Ih=μ0,

where *μ*_1_ is the maximum per capita recovery rate due to the sufficient health care resources and few infectious individuals, *μ*_0_ is the minimum per capita recovery rate due to the basic clinical resources, and *b* is the hospital bed-population ratio which is a nonnegative constant.

Due to the above assumptions, we can formulate the system as follows:

(5) dSpdt=rpSp1−SpKp−βpSpIp,dIpdt=βpSpIp−μp+δpIp,dShdt=Λ−βhShIp1+aIh−μhSh,dEhdt=βhShIp1+aIh−μh+ωhEh,dIhdt=ωhEh−μh+δhIh−μ0+bμ1−μ0b+IhIh,dRhdt=μ0+bμ1−μ0b+IhIh−μhRh.

Detailed descriptions of system parameters and their estimated values are listed in Table 1. The variable *R*_*h*_ can be decoupled from the first four equations of system. Hence, we can reduce system (5) to the following system:

(6) dSpdt=rpSp1−SpKp−βpSpIp,dIpdt=βpSpIp−μp+δpIp,dShdt=Λ−βhShIp1+aIh−μhSh,dEhdt=βhShIp1+aIh−μh+ωhEh,dIhdt=ωhEh−μh+δhIh−μ0+bμ1−μ0b+IhIh.

For system (6), we first show the following result.

**Lemma 1 .**

The set Γ≐{(*S*_*p*_, *I*_*p*_, *S*_*h*_, *E*_*h*_, *I*_*h*_) ∈ *R*_+_^5^ : *S*_*h*_ + *E*_*h*_ + *I*_*h*_ ≤ Λ/*μ*_*h*_} is a positively invariant and attracting region of system (6).

**Proof**

For system (6) with nonnegative initial conditions, the following holds:

(7) dSpdtSp=0=0,dIpdtIp=0=0,dShdtSh=0=Λ>0;

hence, the solutions of *S*_*p*_, *I*_*p*_, and *S*_*h*_ are nonnegative. Then we get

(8) dEhdtEh=0=βhShIp1+aIh≥0,dIhdtIh=0=ωhEh≥0,

so all solutions of system (6) are nonnegative.

Let *N*_*h*_ = *S*_*h*_ + *I*_*h*_ + *E*_*h*_, and it follows that

(9) dNhdt=Λ−μhNh−δhIh−μ0+bμ1−μ0b+IhIh≤Λ−μhNh,

which implies that

(10) $$\begin{array}{c} \underset{t\to +\infty }{\lim}N_{h}\left(\;t\;\right)=\frac{\Lambda }{\mu_{h}}. \end{array}$$

Moreover, if *N*_*h*_(*t*) > Λ/*μ*_*h*_, we have

(11) $$\begin{array}{c} \frac{dN_{h}}{dt}\leq \Lambda -\mu_{h}N_{h}<0. \end{array}$$

Therefore, each solution of system (6) with nonnegative initial conditions initiating from Γ will remain in Γ for *t* > 0.

## 3. Analysis of Equilibria

### 3.1. Existence of Equilibria

In this section, we study the existence of equilibria of system (6) in Γ. By setting the right-hand side of system (6) to zero, we obtain the following equations:

(12) rpSp1−SpKp−βpSpIp=0,βpSpIp−μp+δpIp=0,Λ−βhShIp1+aIh−μhSh=0,βhShIp1+aIh−μh+ωhEh=0,ωhEh−μh+δhIh−μ0+bμ1−μ0b+IhIh=0.

Therefore, the coordinates of equilibria are determined by nonnegative solutions of equations (12). Simple calculation yields that system (6) always has two equilibria $E_{01}(0,0,{\bar{S}}_{h},0,0)$ and $E_{02}(K_{p},0,{\bar{S}}_{h},0,0)$, where ${\bar{S}}_{h}=\Lambda /\mu_{h}$ for all parameter values. We call *E*_01_ and *E*_02_ disease-free equilibria, which represent the state that there is no infection. Using the method proposed by Diekmann et al. [19] and van den Driessche and Watmough [20], the basic reproduction number *R*_0_ of system (6), which is the dominant eigenvalue of the next-generation matrix, can be given by

(13) R0=ρβpKp00βhΛμh00000μp+δp000μh+ωh00−ωhμh+δh+μ1−1=βpKpμp+δp,

where *ρ* is the spectral radius of a matrix.

Next, we discuss the endemic equilibrium denoted by *E*^*∗*^(*S*_*p*_^*∗*^, *I*_*p*_^*∗*^, *S*_*h*_^*∗*^, *E*_*h*_^*∗*^, *I*_*h*_^*∗*^). From a straightforward calculation of the first and second equations of (12), we have

(14) Sp∗=μp+δpβp,

(15) Ip∗=rpβpR0R0−1.

Obviously, if *R*_0_ > 1, *I*_*p*_^*∗*^ is positive. From the last three equations of (12), the coordinates of point *E*^*∗*^ must satisfy

(16) Sh=Λ−μh+ωhEhμh,Eh=μh+δh+μ0Ih+bμ1−μ0/b+IhIhωh.

Substituting (16) into equation *dE*_*h*_/*dt* = 0, after some calculations we have the following equation of *I*_*h*_:

(17) $$\begin{array}{c} f\left(\;I_{h}\;\right)=m_{3}I_{h}^{3}+m_{2}I_{h}^{2}+m_{1}I_{h}+m_{0}, \end{array}$$

where

(18) m3=−ad0μh+ωhωh<0,m2=−d0βhIp∗μh+ωhμhωh−abd1+d0μh+ωhωh<0,m1=ΛβhμhIp∗−bd1βhIp∗μh+ωhμhωh−bd1μh+ωhωh,m0=bβhIp∗Λμh>0.

And *d*_*i*_ = *μ*_*h*_ + *δ*_*h*_ + *μ*_*i*_, *i* = 0,1, and obviously

(19) f0=m0>0,f+∞<0.

Intermediate Value Theorem indicates that there exists at least one positive root of (17). In the following, we consider all the situations.

(i) Assuming there are three real roots, Vieta Theorem indicates that

(20) Ih1Ih2Ih3=−m0m3>0,

(21) Ih1+Ih2+Ih3=−m2m3<0.

From (20) we can see that there are two cases; one is that all three roots are positive and the other is one positive and two negative roots. Equation (21) indicates that at least one root should be negative; hence, in this case, equation (21) has a unique positive root.

(ii) Otherwise, suppose that there are a pair of complex roots and a positive real root, denoted by *x* + *yi*, *x* − *yi*, and *z*, where *x*, *y*, *z* are real numbers. We have

(22) f1=Ih−x+yiIh−x−yiIh−z=Ih3−2x+zIh2+x2+y2+2xzIh−zx2+y2.

Comparing with (17), −*z*(*x*^2^ + *y*^2^) < 0 holds, which contradicts with *m*_0_ > 0.

In summary, we can conclude that (17) only has a unique positive root denoted by *I*_*h*_^*∗*^. Thus there is only one endemic equilibrium *E*^*∗*^(*S*_*p*_^*∗*^, *I*_*p*_^*∗*^, *S*_*h*_^*∗*^, *E*_*h*_^*∗*^, *I*_*h*_^*∗*^) of system (6). The results are listed in the following lemma.

**Lemma 2 .**

In system (6) two disease-free equilibria $E_{01}(0,0,{\bar{S}}_{h},0,0)$ and $E_{02}(K_{p},0,{\bar{S}}_{h},0,0)$ exist, where ${\bar{S}}_{h}=\Lambda /\mu_{h}$ if *R*_0_ < 1 and a unique endemic equilibrium *E*^*∗*^(*S*_*p*_^*∗*^, *I*_*p*_^*∗*^, *S*_*h*_^*∗*^, *E*_*h*_^*∗*^, *I*_*h*_^*∗*^) if *R*_0_ > 1.

### 3.2. The Dynamical Behavior of the Poultry-Only Subsystem

In order to better discuss the full system, we first learn the poultry-only subsystem in *Ω* = {(*S*_*p*_, *I*_*p*_) ∈ *R*_+_^2^ : *S*_*p*_ ≥ 0, *I*_*p*_ ≥ 0},

(23) dSpdt=rpSp1−SpKp−βpSpIp,dIpdt=βpSpIp−μp+δpIp.

Clearly, the poultry-only subsystem (23) is independent of the full system (6). From Lemma 2, we can directly obtain two disease-free equilibria of (23), denoted by *P*_01_(0,0) and *P*_02_(*K*_*p*_, 0) and a unique endemic equilibrium, denoted by *P*^*∗*^(*S*_*p*_^*∗*^, *I*_*p*_^*∗*^) if *R*_0_ > 1.

Linearizing the subsystem (23) at the equilibria *P*_01_, *P*_02_, and *P*^*∗*^, respectively, we can obtain the Jacobian matrices. For *P*_01_, the characteristic equation always has a positive root *r*_*p*_. For *P*_02_, the characteristic equation has two negative roots *λ*_1_ = −*r*_*p*_, *λ*_2_ = (*μ*_*p*_ + *δ*_*p*_)(*R*_0_ − 1) if *R*_0_ < 1. Otherwise it has one positive root. If *R*_0_ > 1, *P*^*∗*^ exists and the characteristic equation is *λ*^2^ + *r*_*p*_(*S*_*p*_^*∗*^/*K*_*p*_)*λ* + *β*_*p*_^2^*S*_*p*_^*∗*^*I*_*p*_^*∗*^ = 0. All roots of the equation have negative real parts. Hence, we summarize the results as follows.

**Lemma 3 .**

The disease-free equilibrium *P*_01_(0,0) is always unstable. Further, (i) if *R*_0_ < 1, the disease-free equilibrium *P*_02_(*K*_*p*_, 0) is locally asymptotically stable and (ii) if *R*_0_ > 1, the disease-free equilibrium *P*_02_(*K*_*p*_, 0) is unstable and the endemic equilibrium *P*^*∗*^(*S*_*p*_^*∗*^, *I*_*p*_^*∗*^) exists and is locally asymptotically stable.

The following theorem shows the global stability of the equilibria.

**Theorem 4 .**

If *R*_0_ < 1, the disease-free equilibrium *P*_02_ is globally asymptotically stable in *R*_+_^2^; if *R*_0_ > 1 the endemic equilibrium *P*^*∗*^ is globally asymptotically stable in *R*_+_^2^.

**Proof**

If *R*_0_ < 1, construct Lyapunov function

(24) $$\begin{array}{c} V_{1}=K_{p}\left(\;\frac{S_{p}}{K_{p}}-\ln\frac{S_{p}}{K_{p}}\;\right)+I_{p}. \end{array}$$

Calculate the derivative *V*_1_ along subsystem (23); it yields

(25) V1′23=KpSp′Kp−Sp′Sp+Ip′=rp1−SpKpSp−βpSpIp−rp1−SpKpKp+βpIpKp+βpSpIp−μp+δpIp=−rpKpSp−Kp2+Ipμp+δpR0−1≤0.

The set *V*_1_′ = 0 has a unique point *P*_02_. According to the invariance principle of Lasalle, all solutions of subsystem (23) approach the largest positively invariant subset of the set *V*_1_′ = 0. Hence, if *R*_0_ < 1, *P*_02_ is globally asymptotically stable in *R*_+_^2^.

If *R*_0_ > 1, consider the Lyapunov function

(26) $$\begin{array}{c} V_{2}=S_{p}^{*}\left(\;\frac{S_{p}}{S_{p}^{*}}-\ln\frac{S_{p}}{S_{p}^{*}}\;\right)+I_{p}^{*}\left(\;\frac{I_{p}}{I_{p}^{*}}-\ln\frac{I_{p}}{I_{p}^{*}}\;\right) \end{array}$$

in *R*_+_^2^. Calculate the derivative *V*_2_ along subsystem (23); it satisfies

(27) V2′23=Sp∗Sp′Sp∗−Sp′Sp+Ip∗Ip′Ip∗−Ip′Ip=rp1−SpKpSp−rp1−SpKpSp∗−rp1−Sp∗KpSp+rp1−Sp∗KpSp∗=−rpKpSp∗−Sp2≤0.

The set *V*_2_′ = 0 has a unique point *P*^*∗*^. According to the invariance principle of Lasalle, all solutions of subsystem (23) approach the largest positively invariant subset of the set *V*_2_′ = 0. Hence, if *R*_0_ > 1, *P*^*∗*^ is globally asymptotically stable in *R*_+_^2^.

### 3.3. The Dynamical Behavior of System (6)

In this section, we will discuss the dynamical behavior of system (6) and study the local stability of equilibria *E*_01_, *E*_02_, and *E*^*∗*^. First, we present the following results.

**Lemma 5 .**

The disease-free equilibrium $E_{01}(0,0,{\bar{S}}_{h},0,0)$ is always unstable. Further, (i) if *R*_0_ < 1, the disease-free equilibrium $E_{02}(K_{p},0,{\bar{S}}_{h},0,0)$ is locally asymptotically stable and (ii) if *R*_0_ > 1, the disease-free equilibrium $E_{02}(K_{p},0,{\bar{S}}_{h},0,0)$ is unstable and the endemic equilibrium *E*^*∗*^(*S*_*p*_^*∗*^, *I*_*p*_^*∗*^, *S*_*h*_^*∗*^, *E*_*h*_^*∗*^, *I*_*h*_^*∗*^) exists and is locally asymptotically stable.

**Proof**

(i) The Jacobian matrix at *E*_01_ is

(28) JE01=rp00000−μp+δp0000−βhΛμh−μh000βhΛμh0−μh+ωh0000ωh−d1.

Since the characteristic equation always has a positive root *λ* = *r*_*p*_, *E*_01_ is always unstable.

(ii) The Jacobian matrix at *E*_02_ is

(29) JE02=−rp−βpKp0000βpKp−μp+δp0000−βhΛμh−μh000βhΛμh0−μh+ωh0000ωh−d1.

One root of the characteristic equation is *λ* = (*μ*_*p*_ + *δ*_*p*_)(*R*_0_ − 1), and others are negative roots. Obviously if *R*_0_ < 1, disease-free equilibrium *E*_02_ is locally asymptotically stable; otherwise *E*_02_ is unstable.

(iii) The Jacobian matrix at *E*^*∗*^ is

(30) $$\begin{array}{c} J\left(\;E^{*}\;\right)=\left[\begin{array}{ccccc} -r_{p}\frac{S_{p}^{*}}{K_{p}} & -\beta_{p}S_{p}^{*} & 0 & 0 & 0\\ \beta_{p}I_{p}^{*} & 0 & 0 & 0 & 0\\ 0 & -\beta_{h}\frac{S_{h}^{*}}{1+aI_{h}^{*}} & -I_{p}^{*}\frac{\beta_{h}}{1+aI_{h}^{*}}-\mu_{h} & 0 & a\beta_{h}I_{p}^{*}\frac{S_{h}^{*}}{{\left(1+aI_{h}^{*}\right)}^{2}}\\ 0 & \beta_{h}\frac{S_{h}^{*}}{1+aI_{h}^{*}} & \beta_{h}\frac{I_{p}^{*}}{1+aI_{h}^{*}} & -\left(\mu_{h}+\omega_{h}\right) & -a\beta_{h}I_{p}^{*}\frac{S_{h}^{*}}{{\left(1+aI_{h}^{*}\right)}^{2}}\\ 0 & 0 & 0 & \omega_{h} & -d_{0}-b^{2}\frac{\left(\mu_{1}-\mu_{0}\right)}{{\left(b+I_{h}^{*}\right)}^{2}} \end{array}\right]. \end{array}$$

The characteristic equation reads

(31) wλ=λ2+rpSp∗Kpλ+βp2Sp∗Ip∗λ3+η2λ2+η1λ+η0=0,

where

(32) η2=βhIp∗1+aIh∗+2μh+ωh+d0+b2μ1−μ0b+Ih∗2>0,η1=βhIp∗1+aIh∗+μh+d0+b2μ1−μ0b+Ih∗2μh+ωh+μh+βhIp∗1+aIh∗d0+b2μ1−μ0b+Ih∗2+aωhβhSh∗Ip∗1+aIh∗2>0,η0=μh+βhIp∗1+aIh∗μh+ωhd0+b2μ1−μ0b+Ih∗2+aμhωhβhSh∗Ip∗1+aIh∗2>0.

It follows that

(33) η2η1−η0=βhIp∗1+aIh∗+μh+μh+⋯·d0+b2μ1−μ0b+Ih∗2μh+ωh+aωhβhSh∗Ip∗1+aIh∗2+⋯−μh+βhIp∗1+aIh∗·μh+ωhd0+b2μ1−μ0b+Ih∗2−aμhωhβh·Sh∗Ip∗1+aIh∗2>0.

By the Routh-Hurwitz criterion, the roots of (31) have negative real parts. The next theorem shows the global dynamics of the system.

**Theorem 6 .**

If *R*_0_ < 1, the disease-free equilibrium *E*_02_ is globally asymptotically stable in Γ; if *R*_0_ > 1 and *b* ≥ 2Λ(*μ*_1_ − *μ*_0_ − (*μ*_*h*_/2))/*μ*_*h*_^2^ > 0, the endemic equilibrium *E*^*∗*^ is globally asymptotically stable in Γ.

**Proof**

If *R*_0_ < 1, Theorem 4 indicates that the disease-free equilibrium *P*_02_(*K*_*p*_, 0) is globally asymptotically stable in subsystem (23). By calculation, system (6) can be reduced to the following system:

(34) dShdt=Λ−μhSh,dEhdt=−μh+ωhEh,dIhdt=ωhEh−μh+δhIh−μ0+bμ1−μ0b+IhIh.

From the first two equations of system (34), we can obtain

(35) Sht=Sh0−Λμhexp⁡−μht+Λμh,Eht=Eh0exp⁡−μh+ωht.

Clearly, we have that

(36) limt→+∞⁡Sh=S¯h,limt→+∞⁡Eh=0,

further, since

(37) $$\begin{array}{c} \frac{dI_{h}}{dt}=-d_{0}I_{h}-\frac{b\left(\mu_{1}-\mu_{0}\right)I_{h}}{\left(b+I_{h}\right)}<0; \end{array}$$

that is, *I*_*h*_(*t*) is a monotonically decreasing function and lim_*t*→+*∞*_⁡*I*_*h*_ = 0. In summary, the disease-free equilibrium *E*_02_ is globally asymptotically stable.

If *R*_0_ > 1, Theorem 4 indicates that the endemic equilibrium *P*^*∗*^(*S*_*p*_^*∗*^, *I*_*p*_^*∗*^) is globally asymptotically stable in subsystem (23). Similarly, we can also simplify system (6) as

(38) dShdt=Λ−βhShIp∗1+aIh−μhSh,dEhdt=βhShIp∗1+aIh−μh+ωhEh,dIhdt=ωhEh−μh+δhIh−μ0+bμ1−μ0b+IhIh.

From the Jacobian matrix of system (38), we can obtain the second additive compound matrix **J**^[2]^:

(39) J2=−j11−aβhShIp∗1+aIh2−aβhShIp∗1+aIh2ωh−j2200βhIp∗1+aIh−j33,

where

(40) j11=βhIp∗1+aIh+2μh+ωh,j22=βhIp∗1+aIh+μh+d0+b2μ1−μ0b+Ih2,j33=μh+ωh+d0+b2μ1−μ0b+Ih2.

We choose the matrix **P**(*S*_*h*_, *E*_*h*_, *I*_*h*_) = diag⁡(1, *E*_*h*_/*I*_*h*_, *E*_*h*_/*I*_*h*_) and calculate **P**_*f*_, which denotes the matrix whose components are **P**_*f*_*ij*__(*x*) = (∂**P**_*ij*_(*x*)/∂*x*)^*T*^ · *f*(*x*), so **P**_*f*_**P**^−1^ = diag⁡(0, *E*_*h*_′/*E*_*h*_ − *I*_*h*_′/*I*_*h*_, *E*_*h*_′/*E*_*h*_ − *I*_*h*_′/*I*_*h*_). Rewrite the matrix **B** = **P**_*f*_**P**^−1^ + **PJ**^[2]^**P**^−1^ in block matrix

(41) $$\begin{array}{c} B=\left[\begin{array}{cc} B_{11} & B_{12}\\ B_{21} & B_{22} \end{array}\right], \end{array}$$

where

(42) B11=−j11,B12=−aβhShIp∗1+aIh2IhEh,−aβhShIp∗1+aIh2IhEh,B21=ωhEhIh,0T,B22=−j22+Eh′Eh−Ih′Ih0βhIp∗1+aIh−j33+Eh′Eh−Ih′Ih.

We consider the norm ‖·‖ in *R*_3_^+^ as

(43) $$\begin{array}{c} \left\|\;\left(\;S_{h},E_{h},I_{h}\;\right)\;\right\|=\max\left\{\;\left|\;S_{h}\;\right|,\left|\;E_{h}\;\right|+\left|\;I_{h}\;\right|\;\right\}, \end{array}$$

with vector (*S*_*h*_, *E*_*h*_, *I*_*h*_) in *R*_3_^+^ and denote by *μ*(**B**) the Lozinskil measure with respect to this norm. It follows that

(44) μB≤sup⁡g1,g2≡sup⁡μ1B11+B12,B21+μ1B22,

where |**B**_12_|, |**B**_21_| are matrix norms with respect to the *L*^1^ vector norm and *μ*_1_ denotes the Lozinskil measure with respect to the *L*^1^ norm.

We calculate *g*_1_ = *μ*_1_(**B**_11_) + |**B**_12_|, where

(45) μ1B11=−j11,B12=aβhShIp∗1+aIh2IhEh.

Hence,

(46) g1=−j11+aβhShIp∗1+aIh2IhEh=−βhIp∗1+aIh−2μh−ωh+aβhShIp∗1+aIh2IhEh.

Further, *g*_2_ = |**B**_21_| + *μ*_1_(**B**_22_), where

(47) μ1B22=max−j22+Eh′Eh−Ih′Ih+βhIp∗1+aIh,−j33+Eh′Eh−Ih′Ih=max−μh−d0−b2μ1−μ0b+Ih2+Eh′Eh−Ih′Ih,−μh−ωh−d0−b2μ1−μ0b+Ih2+Eh′Eh−Ih′Ih=−μh−d0−b2μ1−μ0b+Ih2+Eh′Eh−Ih′Ih,

and, hence,

(48) $$\begin{array}{c} g_{2}=-\mu_{h}-d_{0}-b^{2}\frac{\left(\mu_{1}-\mu_{0}\right)}{{\left(b+I_{h}\right)}^{2}}+\frac{E_{h}^{\prime }}{E_{h}}-\frac{I_{h}^{\prime }}{I_{h}}+\omega_{h}\frac{E_{h}}{I_{h}}. \end{array}$$

From the last two equations of system (38), we have

(49) Ih′Ih+d0−ωhEhIh=−bμ1−μ0b+Ih,Eh′Eh−βhShEhIp∗1+aIh=−ωh+μh.

Taking into consideration (49), the following holds:

(50) g1=Eh′Eh−μh−βhIp∗1+aIh−βhIp∗1+aIh2ShEh,g2=Eh′Eh−μh+bμ1−μ0b+Ih2Ih,

and thus

(51) μB≤sup⁡g1,g2≤Eh′Eh−μh+bμ1−μ0b+Ih2Ih≤Eh′Eh−μh+Ihμ1−μ0b+Ih.

We assume *b* ≥ 2Λ(*μ*_1_ − *μ*_0_ − *μ*_*h*_/2)/*μ*_*h*_^2^ > 0; hence

(52) $$\begin{array}{c} \mu \left(\;B\;\right)\leq \frac{E_{h}^{\prime }}{E_{h}}-\frac{\mu_{h}}{2}, \end{array}$$

and then

(53) q=lim supt→+∞ ⁡supx∈Γ⁡1t∫0tμBds≤1t∫0t∗μBds+1tln⁡EhtEht∗−μh2t−t∗t<0.

The Bendixson condition is satisfied; then the result follows.

## 4. Numerical Simulations

In this section, we carry out numerical simulations for system (5) in order to illustrate the influences of the basic reproduction number *R*_0_, psychological effect, and hospital resources on the disease evolution. The lifespan of poultry for chickens is 5 to 10 years under favorable conditions [21]; thus we assume the poultry can survive 8 years and fix the parameter *μ*_*p*_ = 3.4246*∗*10^−4^. People can usually live for 70 years, so the natural death rate of human *μ*_*h*_ is 3.91*∗*10^−5^. The latent period is about 7 days (China CDC) and *ω*_*h*_ = 1/7. We assume the following parameters: *r*_*p*_ = 5*∗*10^−3^, *K*_*p*_ = 5*∗*10^4^, *δ*_*p*_ = 4*∗*10^−4^, Λ = 30, *β*_*h*_ = 7*∗*10^−9^, *μ*_0_ = 0.067, *δ*_*h*_ = 0.077. We choose the initial values as (*S*_*p*_(0), *I*_*p*_(0), *S*_*h*_(0), *E*_*h*_(0), *I*_*h*_(0), *R*_*h*_(0)) = (1000000,500,100000,3, 1,0).

**Figure 1:**
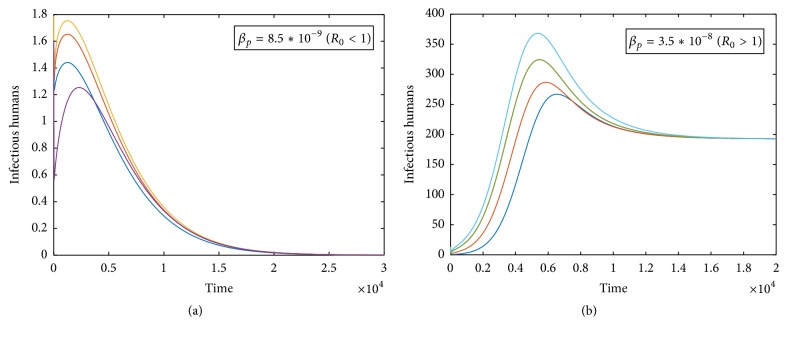
(a) All solutions of *I*_*h*_(*t*) converge to the disease-free steady state eventually if *β*_*p*_ < *β*_*p*_^*∗*^  (*R*_0_ < 1). (b) All solutions of *I*_*h*_(*t*) converge to the endemic steady state eventually if *β*_*p*_ > *β*_*p*_^*∗*^  (*R*_0_ > 1).

Our theoretical results show that the basic reproduction number *R*_0_ determines the global dynamics of the system (5). Fix *a* = 0.001, *b* = 0.05, and *μ*_1_ = 0.1, for *R*_0_ = *β*_*p*_*K*_*p*_/(*μ*_*p*_ + *δ*_*p*_); when *R*_0_ equals 1, we obtain *β*_*p*_^*∗*^ = 1.48*∗*10^−8^. If *β*_*p*_ < *β*_*p*_^*∗*^  (*R*_0_ < 1), the solutions of *I*_*h*_ converge to the disease-free steady state and the disease will finally be extinct (see Figure 1(a)). If *β*_*p*_ > *β*_*p*_^*∗*^  (*R*_0_ > 1), the solutions of *I*_*h*_ converge to the endemic state, which implies that the disease will persist (see Figure 1(b)).

**Figure 2:**
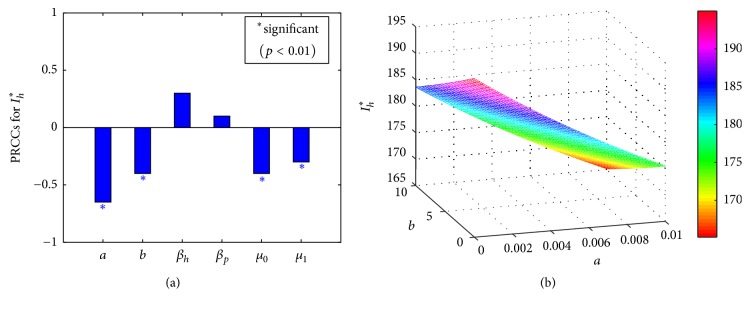
(a) PRCCs for the endemic equilibrium prevalence. All the parameters came from Latin hypercube sampling. (b) Plot of the endemic equilibrium prevalence with respect to the psychological effect parameter *a* and hospital bed ratio *b*. *β*_*p*_ = 3.5*∗*10^−8^  (*R*_0_ > 1), *μ*_1_ = 0.24.

We then use Latin hypercube sampling (LHS) [22] and partial rank correlation coefficients (PRCCs) [23] to explore parameter space and find to which parameter the prevalence at endemic equilibrium is sensitive when parameters vary. Due to limited data on the distribution for each parameter, we choose a uniform distribution for all input parameters with the mean value listed in Table 1. PRCC results in Figure 2(a) indicate that the first four parameters with the most significant impact on the equilibrium prevalence are the psychological effect parameter *a*, the hospital bed-population ratio *b*, the minimum recovery rate of human *μ*_0_, and maximum recovery rate of human *μ*_1_. It is reasonable that the four parameters play important roles in the infections. In fact, a larger psychological effect parameter *a* means that the public improve their awareness of A(H7N9) virus and take more preventive measures, which leads to lower incidence rate and then lower new infections. A larger hospital bed ratio *b* indicates that more sufficient hospital resources and treatments are provided, which then can improve the recovery rate and lead to lower new infections. The results can be seen explicitly from Figure 2(b). When the impact of psychological effect and hospital resources is introduced, the amount of equilibrium prevalence obviously decreases with the parameters *a* and (or) *b* increasing.

**Figure 3:**
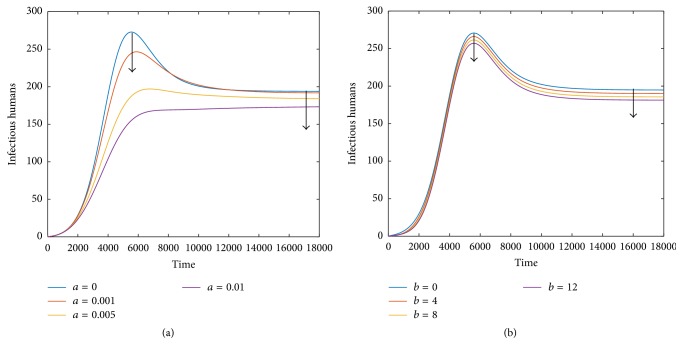
Fix *β*_*p*_ = 3.5*∗*10^−8^  (*R*_0_ > 1). (a) Plot of *I*_*h*_ with varying parameter *a* for *b* = 1. (b) Plot of *I*_*h*_ with varying parameter *b* for *a* = 0.001. In both cases, the final size and the peak value of the infected are diminished.

To further examine the impact of psychological effect and hospital resources on infections, respectively, we take *β*_*p*_ = 3.5*∗*10^−8^  (*R*_0_ = 2.3570 > 1) and *μ*_1_ = 0.24 with one of parameters *a* and *b* fixed and the other varying.  Figure 3(a) shows that slightly increasing parameter *a* can not only diminish the final size of the infected but also result in a much lower peak of the disease. Similar results can be obtained when parameter *b* varies (see Figure 3(b)).

## 5. Conclusions and Discussions

In this work, in order to evaluate the combined impact of psychological effect and available hospital resources on the transmission of A(H7N9) virus from poultry to humans, we formulated and analyzed a dynamical model with a nonlinear incidence rate and a nonlinear recovery rate. From the mathematical point of view, we obtained the basic reproduction number *R*_0_, which determines the extinction of the avian influenza. Theoretical analysis of system (6) indicates that the disease-free equilibrium $E_{02}(K_{p},0,{\bar{S}}_{h},0,0)$ is globally asymptotically stable in Γ when the basic reproduction number is less than unity; that is, the avian influenza A(H7N9) will die out (see Figure 1(a)); and the endemic equilibrium *E*^*∗*^(*S*_*p*_^*∗*^, *I*_*p*_^*∗*^, *S*_*h*_^*∗*^, *E*_*h*_^*∗*^, *I*_*h*_^*∗*^) is globally asymptotically stable in Γ when the basic reproduction number is larger than unity and *b* ≥ 2Λ(*μ*_1_ − *μ*_0_ − *μ*_*h*_/2)/*μ*_*h*_^2^ > 0. Note that although the global stability of endemic equilibrium is obtained under this specific condition, which may be due to the limitations of the analytical method, numerical simulations show that all solutions can converge to *E*^*∗*^ eventually without the specific condition (see Figure 1(b)).

Both the psychological effect and available hospital resources cannot neither change the stability of endemic equilibrium nor alter the basic reproduction number, but they indeed play a significant role in affecting the number of infectious humans, seen from PRCC results (Figure 2(a)) and the impact of parameters *a* and *b* on equilibrium prevalence (Figure 2(b)). Comparing the number of infectious humans with or without psychological effects, that is, parameter *a* = 0 or *a* > 0, it can be seen that bigger parameter *a* can significantly decrease the peak of A(H7N9) infections; meanwhile, the final size of the disease can be reduced. However, no matter whether there is psychological effect or not, the disease cannot die out, seen from Figure 3(a). Figure 3(b) indicates that when the available hospital resources are more sufficient, a bigger parameter *b* leads to a smaller size of the outbreak and a lower number of infectious humans. Similarly, the impact of available hospital resources cannot eradicate the disease either.

Different from the previous avian influenza dynamics models, which usually use bilinear and standard incidence rates and constant recovery rate, in this work, incorporating the combined impact of psychological effect and available hospital resources, we formulate A(H7N9) dynamic model with nonlinear incidence rate and nonlinear recovery rate. We introduce the recovery function *μ*(*b*, *I*_*h*_) = *μ*_0_ + *b*(*μ*_1_ − *μ*_0_)/(*b* + *I*_*h*_), where parameter *b* represents hospital bed-population ratio, which reflects the available resources of the health care system to public. The number of hospital beds is a critical index and with the number of infected cases increasing it may become a limiting factor in controlling the spread of A(H7N9) virus. Our results demonstrate that both psychological effect and available hospital resources can dramatically affect the A(H7N9) virus transmission dynamics. This work is an improvement of existing models of the avian influenza A(H7N9) and the results can provide some practical implications for the control of A(H7N9) virus transmission.

Note that, from current data for A(H7N9) infection, there is an incubation period between infection and symptom onset in both avian and human populations [24]. We consider latent class (*E*_*h*_) in our model, which is more realistic to exhibit the epidemiology of A(H7N9). Based on this characteristic of A(H7N9) virus, we will incorporate time delay in our model for future study. There have been five seasonal outbreaks of human infection by A(H7N9) virus in China, since the first outbreak was observed in 2013. Except for the first outbreak, others usually started in October, significantly increased in late December, and then peaked in January of the next year [25]. Thus seasonal variation may affect the spread of A(H7N9) virus as one of the important factors. Zhao et al. [26] presented a model with period parameters to analyze the effect of climate change on the transmission of A(H7N9) and discussed the global stability and threshold conditions. In our future work, we can also consider the incidence rate as a periodic function.

## Figures and Tables

**Table 1:** Description of parameters.

Parameter	Description	Value	Reference
*r*_*p*_	Intrinsic growth rate of poultry	5 × 10^−3^	[6]
*K*_*p*_	Maximal carrying capacity of the poultry	50000	[6]
*β*_*p*_	Transmission rate from infectious poultry to susceptible poultry	-	-
*μ*_*p*_	Natural death rate of poultry (chicken)	1/5–1/10 year^−1^	[21]
*δ*_*p*_	Disease induced death rate of poultry	4 × 10^−4^	[6]
Λ	New recruitment and newborn of human	30	[6]
*β*_*h*_	Transmission rate from infectious poultry to susceptible human	5 × 10^−9^	Assumed
*μ*_*h*_	Natural death rate of human	1/70 year^−1^	Assumed
*δ*_*h*_	Disease induced death rate of human	0.077	[27]
*ω*_*h*_	Progression to latent rate of human	1/7 day^−1^	CDC
*μ*_0_	Minimum recovery rate of human	(0.067–0.100)	[27]
*μ*_1_	Maximum recovery rate of human	(*μ*_0_, 10)	[18]
*b*	Hospital bed-population ratio	(0,20)	[18]
*a*	Psychological effect parameter	-	-
